# Non-Small Cell Lung Cancer Targeted Therapy: Drugs and Mechanisms of Drug Resistance

**DOI:** 10.3390/ijms232315056

**Published:** 2022-12-01

**Authors:** Jiajia Wu, Zhenghong Lin

**Affiliations:** School of Life Sciences, Chongqing University, Chongqing 401331, China

**Keywords:** NSCLC, targeted therapy, drug resistance, molecular mechanisms, EGFR

## Abstract

The advent of precision medicine has brought light to the treatment of non-small cell lung cancer (NSCLC), expanding the options for patients with advanced NSCLC by targeting therapy through genetic and epigenetic cues. Tumor driver genes in NSCLC patients have been uncovered one by one, including epidermal growth factor receptor (EGFR), mesenchymal lymphoma kinase (ALK), and receptor tyrosine kinase ROS proto-oncogene 1 (ROS1) mutants. Antibodies and inhibitors that target the critical gene-mediated signaling pathways that regulate tumor growth and development are anticipated to increase patient survival and quality of life. Targeted drugs continue to emerge, with as many as two dozen approved by the FDA, and chemotherapy and targeted therapy have significantly improved patient prognosis. However, resistance due to cancer drivers’ genetic alterations has given rise to significant challenges in treating patients with metastatic NSCLC. Here, we summarized the main targeted therapeutic sites of NSCLC drugs and discussed their resistance mechanisms, aiming to provide new ideas for follow-up research and clues for the improvement of targeted drugs.

## 1. Introduction

**Figure 1:**
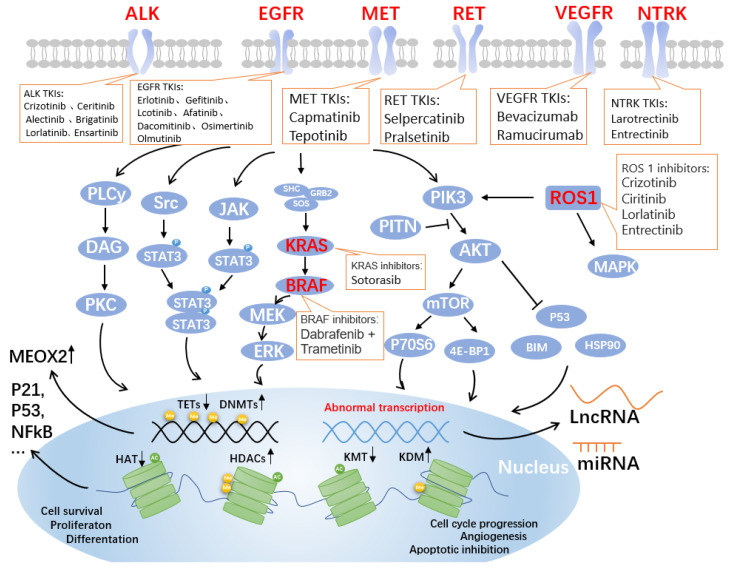
Genes and pathways associated with targeted drugs for NSCLC. Four critical signaling pathways include JAK-STAT, MAPK, PLC-gamma (phospho-lipase C gamma), and PI3K-AKT. These pathways are well-known controllers of cell cycle progression, proliferation, and apoptosis/cell survival; deregulation is a frequent characteristic of human malignancies. Alterations in key pathways will affect DNA methylation modifications, such as increased DNA methyltransferases (DNMTs) and decreased the ten-eleven translocation methylcytosine dioxygenases (TETs), further allowing overexpression of mesenchymal homology box 2 (MEOX2), whose expression is negatively correlated with patient survival. Additionally, post-translational histone modifications were affected. As shown in the figure, histone acetyltransferases (HATs), histone deacetylases (HDACs, also known as lysine deacetylases or KDACs), the lysine methyltransferases (KMTs) and lysine demethylases (KDMs) undergo corresponding up- or downregulation, affecting the expression of P21, P53, nuclear factor κB (NFκB), and other related proteins that are closely related to the cell cycle. Non-coding RNAs, such as long non-coding RNAs (LncRNAs) and miRNAs, are produced as a result of abnormal transcription. The lncRNA is a brand-new class of regulatory RNA. The LncRNA HOX antisense intergenic RNA (HOTAIR), an oncogene in NSCLC, is one of the significant factors controlling the growth of malignancies. Unknown are the immunomodulatory pathway and probable molecular mechanism involved in NSCLC. Notably, the graphic labels current FDA-approved medications that target EGFR, ALK, MET, RET, VEGF, NTRK, ROS1, KRAS, and BRAF.

Cancer is the second most significant cause of mortality in the United States and an important global public health issue. In the United States, there are anticipated to be 1,918,030 new cancer cases and 609,360 cancer deaths in 2022, with lung cancer accounting for around 350 of those fatalities daily and being the primary reason for cancer deaths [1]. According to earlier research, lung cancer killed more men and women under 40 and women over 60 than breast, prostate, colorectal, and leukemia combined [2]. With 85% of all new diagnoses, non-small cell lung cancer (NSCLC) is the most prevalent subtype of lung cancer [3]. The dismal five-year survival rate for NSCLC is 15%. The prognosis of patients has dramatically improved thanks to chemotherapy and targeted treatments [4]. Molecular detection has become a mandatory method for the management of NSCLC patients. The investigation of anaplastic lymphoma kinase (ALK), receptor tyrosine kinase ROS proto-oncogene 1(ROS1), rearranged in transfection (RET), and neurotrophic tyrosine receptor kinase (NTRK) translocations, and the identification of epidermal growth factor receptor (EGFR), V-RAF mouse sarcoma virus oncogene homolog B1 (BRAF), and mesenchymal–epithelial transition factor (MET) mutations have already been included in the NSCLC diagnostic standards. These kinase inhibitors are commonly used in clinical practice [5]. The Food and Drug Administration (FDA) has recently approved several medications for the treatment of NSCLC, of which the primary targets for kinase inhibitor therapy in NSCLC have now targeted activation of EGFR, ALK, ROS1, and BRAF, MET, and RET (Figure 1). Other oncogenic driver subtypes of NSCLC are currently being evaluated for targeted therapy [6]. As a result of receptor tyrosine kinase (RTK) activation, the intracellular structural domain of EGFR is autophosphorylated, and the phosphotyrosine residues form to serve as docking points for various adapter molecules, thereby inducing downstream signaling [7]. In turn, the rat sarcoma (RAS)/rapidly accelerated fibrosarcoma (RAF)/mitogen-activated protein kinase (MAPK) pathway, phosphatidylinositol-3 kinase (PI3K)/protein kinase B (AKT) pathway, and Janus kinase (JAK)/signal transducer and activator of transcription (STAT) pathway are activated, which in turn stimulate mitosis, lead to cell proliferation, and inhibit apoptosis [8]. Although significant advances have been made in available therapies for NSCLC, acquired drug resistance remains a significant barrier to the treatment. The ability to cure advanced NSCLC has yet to be achieved, despite our growing understanding of the many oncogenic drivers of this disease. Instead, the emergence of resistance remains the rule [9].

Herein, we summarized the significant targeted therapeutic loci and approved drugs for NSCLC and introduce their molecular mechanisms of drug resistance, which will be helpful for the drug design and subsequent treatment of NSCLC.

## 2. Major Target Sites and Drugs for NSCLC

### 2.1. EGFR-TKIs

The ErbB family of RTKs, which also includes ErbB-1 (HER1, EGFR), ErbB-2 (HER2, Neu), ErbB-3 (HER3), and ErbB-4, comprises the transmembrane glycoprotein known as EGFR (HER4). When EGFR binds to ligands, specific intracellular signaling pathways, including PI3K/Akt and MAPK, which are involved in the proliferation, differentiation, migration, and death of some cells, are stimulated (Figure 1) [10].

Since EGFR is expressed by more than 60% of NSCLCs, it has become a crucial therapeutic target for treating these malignancies. Inhibitors targeting the structural domain of tyrosine kinase inhibitors (TKIs) have been developed and are clinically active. Moreover, these TKIs are especially effective in patients who contain activating mutations in the tyrosine kinase structural domain of the EGFR gene [11].

Erlotinib and gefitinib are examples of first-generation medications that are reversible inhibitors. Erlotinib’s group was shown to have a median progression-free survival (PFS) of 9.7 months, while the group receiving conventional chemotherapy had a median PFS of 5.2 months [12]. Afatinib and dacomitinib are examples of irreversible second-generation inhibitors that bind to EGFR covalently. In contrast to platinum-based chemotherapy, patients with EGFR-mutant cancers showed >70% radiological response times and statistically significantly improved PFS when treated with first-generation (erlotinib and gefitinib) or second-generation (afatinib) EGFR TKIs (Table 1) [10,13,14,15].

For metastatic EGFR-mutant NSCLC patients who have developed the EGFR T790M resistance mutation, osimertinib was the first third-generation EGFR TKI to obtain FDA and EMA approval [16]. For patients with EGFR-mutant NSCLC, osimertinib is superior to erlotinib and gefitinib as the first-line treatment [17].

**Table 1:** Summary of FDA-approved EGFR-TKI.

Generation	Drug	Approval Status	Reversible/Irreversible	Median PFS (Months)	Ref.
1st	Erlotinib	FDA, EMA	Reversible	9.7	[13]
	Gefitinib	FDA, EMA	Reversible	10.8	[14]
	Icotinib	CFDA	Reversible	10	[18]
2nd	Afatinib	FDA, EMA, CFDA	Irreversible	11.0	[19]
	Dacomitinib	FDA	Irreversible	14.7	[20]
3rd	Osimertinib	FDA, MEA	Irreversible	18.9	[21]
	Olmutinib	KFDA (Conditional)	Irreversible	NR	[19]

Abbreviations: EMA, the European Medicines Agency; CFDA, the China Food and Drug Administration; KFDA, the Korea Food and Drug Administration; NR, not reported.

### 2.2. ALK-TKIs

The ALK gene encodes a tyrosine kinase receptor and is located on the short arm of chromosome 2 (2p23), belongs to the insulin receptor superfamily, and encodes for the ALK protein. The oncogenic ALK fusion gene is present in 3–5% of NSCLC patients [22]. ALK is a transmembrane tyrosine kinase receptor that functions similarly to other RTKs in that it has an extracellular domain, a membrane segment, and a cytoplasmic receptor kinase region [23,24]. In NSCLC, more than 19 distinct ALK fusion partners, including EML4, KIF5B, KLC1, and TPR, have been identified [25]. About 85% of all fusion variants in ALK+ NSCLC are represented by the prevalent fusion variant, EML4-ALK. Additionally, the most frequent genetic co-alterations in ALK+ NSCLC are TP53 mutations [25].

The first oral ALK TKI approved for the treatment of non-small cell lung cancer (NSCLC) that was positive for ALK, crizotinib, initially showed promising outcomes (Table 2). The initial euphoria, however, was subdued because almost all of the treated individuals unavoidably developed resistance within a year and experienced disease progression, mainly in the brain or other parenchymal areas [26]. When crizotinib binds to the ATP pocket of the MET kinase in a DFG-in conformation, it forms conventional hydrogen bonds (Hb) with the residues in the hinge area. Additionally, the activation loop and its phenyl ring interact poorly (A-loop). The medication was discovered to have unintended effects on ALK and other kinases [8]. Second-generation ALK TKIs such as crizotinib (LDK378), alectinib (CH5424802/RO5424802), and brigatinib (AP26113) were developed to combat therapy-induced acquired resistance and boost efficacy in ALK-positive patients receiving crizotinib pretreatment, even those with metastases to the central nervous system (CNS) (Table 2). [27,28]. Alectinib forms a classical Hb with M1199 by attaching to the ATP-binding site of ALK. Additionally, alectinib interacts with several additional nearby residues from the -helix (K1150, E1167), the catalytic loop (R1253), and the DFG motif via solvent water molecules (G1269, D1270). As a result, the substance is a part of a stabilizing global Hb network that may likely make up for any one mutation at the binding site [8,29]. Furthermore, third-generation ALK TKIs, including lorlatinib (PF-06463922), entrectinib (RxDx-101), and ensartinib (X-398), provided promising early findings in terms of both clinical activity and safety, according to recent clinical trials (Table 2). [30].

### 2.3. Other Targeted Sites and Drugs for NSCLC

#### 2.3.1. ROS1

The ROS proto-oncogene 1 is a member of the insulin receptor subfamily and is encoded by the ROS1 gene on chromosome 6Q22.1 [35]. It has a sizable hydrophobic single-pass transmembrane region, an extensive N-terminal extracellular structural domain, and a C-terminal intracellular tyrosine kinase structural domain [36]. ROS1 rearrangements, a fusion that encourages tumorigenicity and/or independent growth of different cell lines, are present in 1–2% of NSCLC patients [37,38], and these patients are more likely to be female and to have smoked less [39]. The median age of the 29 individuals with ROS1 rearrangement was 51, ranging from 30 to 80 years old, and 68.9% of them had never smoked [40]. At first glance, the proportion may seem small, but given the massive base of NSCLC patients, it is estimated that there are 10,000–15,000 new cases of the disease worldwide each year [35].

Phylogenetic sequence analysis identified that ROS1 has been linked to the ALK/LTK and insulin receptor RTK families. Homology with ALK is very significant in the development of ROS1-directed medications; nevertheless, not all ALK TKI exhibit dual inhibitory activity against ALK and ROS1. In 2016, the U.S. FDA and the European Medicines Agency approved the drug crizotinib, a multitargeted MET, ALK, and ROS1 inhibitor that showed considerable efficacy in NSCLCs with ROS1 rearrangements in a phase I study [41]. The ROS1 expansion group of crizotinib’s phase I trial had an objective response rate (ORR) of 72%. The overall response duration was 17.6 months, whereas the median PFS was 19.2 months [42]. Four drugs with notable action against ROS1+ NSCLC are FDA-approved: crizotinib, ciritinib, lorlatinib, and entrectinib (Table 3). Entrectinib, lorlatinib, and ciritinib all had an overall response rate of more than 60%, with entrectinib having an intracranial activity [43].

#### 2.3.2. BRAF

The serine/threonine protein kinase family includes mutations in the v-RAF murine sarcoma viral oncogene homolog B (BRAF), a crucial effector molecule for the MAPK/ERK signaling pathway (Figure 1). BRAF mutations are present in 4% of NSCLC, and 50% of these mutations are not V600 variants [44]. By breaking the glycine-rich P loop and its variant domain of the kinase segment, somatic mutations in BRAF that result in the V600E variation change two major areas of the peptide. In WT BRAF, the transition between the active and inactive states is accomplished by activating the inhibitory effect caused by the glycine-rich P loop, which is crucial for incorporating the signal transduction supplied by RAS [45,46]. There is no preference for race in the prevalence of BRAF mutant lung cancer, which ranges from 1.5% to 3.5% [47].

The FDA expanded the use of dabrafenib and trametinib on 22 June 2017, allowing for the treatment of patients with metastatic NSCLC who have the BRAF(V600E) mutation [48]. A two-cohort phase II study compared patients treated with dabrafenib as a single agent with dabrafenib in combination with trametinib and found that the ORR was 33% vs. 67% and the PFS was 5.5 vs. 10.2 months, respectively [49]. Additionally, the French National Cancer Institute (INCA) experiment showed that BRAF(V600E) mutation-positive NSCLC patients responded well to vemurafenib monotherapy, although BRAF(nonV600) mutation-positive individuals did not [50]. For patients with advanced or metastatic melanoma, non-small cell lung cancer, or anaplastic thyroid cancer and BRAF(V600E/K) mutations, the U.S. FDA has currently approved three RAF and MEK inhibitor combinations: vemurafenib/cobimetinib (Genentech, San Francisco, CA, USA), dabrafenib/trametinib (Novartis, Basel, Switzerland), and encorafenib/binimetinib (Array BioPharma, Boulder, CO, USA) [51].

#### 2.3.3. MET

The MET receptor is located on the long arm of human chromosome 7 (7q31) and is encoded by the MET oncogene. This oncogene was first identified in a human osteosarcoma cell line containing the transforming fusion protein TPR–MET, generated by a rearrangement between a translocation promoter region (TPR) located on chromosome 1 at the 5’ end and the MET gene located on chromosome 7 at the 3’ end [52,53]. HGF ligand binding to the MET receptor causes homodimerization and phosphorylation of intracellular tyrosine residues, which activates MET [54]. This triggers the downstream signaling pathways for RAS/ERK/MAPK, PI3K-AKT, Wnt/catenin, and STAT [55].

Small cell lung cancer was the first disease to be linked to somatic mutations affecting splicing sites of exon 14 of the MET gene, which codes for the juxtamembrane region [56]. The median age was 61 years for patients with EGFR mutations, 65 years for KRAS mutant NSCLC, and a significantly older median age of 72.5 years for patients with MET exon 14 mutant NSCLC. Overall, 36% of MET exon 14 mutation patients had never smoked, and 68% were female [57]. At least seven TKIs targeting MET gene mutations are currently on the market or in clinical trials, including crizotinib, cabozantinib, voritinib, tepotinib, capmatinib, glesatinib, and merestinib, with additional drugs in preclinical studies [58]. Tepotinib, capmatinib, and savolitinib have all demonstrated potent actions in phase I/II investigations; in fact, tepotinib and capmatinib were approved for usage by health authorities [59]. Tepotinib and capmatinib received FDA approval on 3 February 2021, and 6 May 2020, respectively. Patients with metastatic non-small cell lung cancer (mNSCLC) whose tumors carry an exon 14 skipping mutation associated with the mesenchymal–epithelial transition (MET) are advised to take capmatinib. Tepotinib is recommended for people with mNSCLC who had MET exon 14 skipping mutations [60].

#### 2.3.4. RET

Transmembrane glycoprotein receptor-tyrosine kinase is produced during transfection by the RET (rearranged during transfection) proto-oncogene, which is located on chromosome 10 [61]. The RET gene can be found in 1% to 2% of all NSCLC patients undergoing chromosomal rearrangement and is involved in various upstream fusion partners, such as KIF5B, TRIM33, CCDC6, and NCOA4 [62]. Multitarget inhibitors with anti-rearranged during RET action have been studied in patients with RET-rearranged lung cancer in several preclinical models, clinical trials, and retrospective investigations to date. The advantage in terms of response (16–47%) and PFS (2–7 months) in the clinical situation is not comparable to that reported with other targeted medicines in NSCLC patients with oncogene addiction [63]. The FDA approved pralsetinib in September 2020 for the treatment of people with metastatic RET fusion-positive NSCLC [64]. This is the first oral tyrosine kinase inhibitor that can be taken once a day by people with metastatic NSCLC that is RET fusion positive. Patients who had received platinum-based chemotherapy in the past or had just started treatment were shown to have response rates of 57% and 70%, respectively, to pralsetinib [65].

#### 2.3.5. KRAS

The proto-oncogene KRAS (Kirsten rat sarcoma 2 viral oncogene homolog) produces the small GTPase transductor protein KRAS [66]. Overall, KRAS accounts for 85% of RAS mutations observed in human cancers, and KRAS(G12C) mutation occurs in 13% of NSCLCs [67]. Although the panorama of treatment for advanced NSCLC has been significantly altered in recent years by the use of targeted therapies and immune checkpoint inhibitors, past attempts to target KRAS (direct and indirect approaches) have not been particularly successful [67]. The RAF-MEK-ERK pathway is one of the cell growth and division pathways that is promoted by KRAS(G12C) mutations [68]. For the treatment of adult patients with locally advanced or metastatic NSCLC with KRAS (G12C) mutations who have undergone at least one prior systemic therapy as established by the FDA-approved test, sotorasib was given accelerated approval by the FDA in May 2021 [69]. In a phase I study, sotorasib demonstrated antitumor effects in patients with advanced solid tumors bearing the KRAS (G12C) mutation. In a single-arm phase II trial, 33.9% of patients had partial remissions and 4.2% had complete remissions, making up the total number of patients who had objective remissions. The average length of remission was 11.1 months [70,71]. Sotorasib, an oral small molecule inhibitor of the RAS GTPase family, irreversibly binds to the P2 pocket of inactive GDP-bound KRAS. The cysteine in KRAS (G12C) establishes an irreversible covalent bond with sotorasib, immobilizing the protein in an inactive state. By preventing KRAS signaling, sotorasib inhibits both in vitro and in vivo cell growth as well as tumor growth, and it only causes apoptosis in KRAS (G12C) tumor cell lines [68,72].

#### 2.3.6. VEGF

The growth factor known as vascular endothelial growth factor (VEGF) has significant pro-angiogenic activity and affects endothelial cells in a mitogenic and anti-apoptotic manner. It also enhances vascular permeability and encourages cell migration. These results mean that it actively contributes to the regulation of both healthy and unhealthy angiogenic processes [73].

The first VEGF inhibitor to be authorized for cancer treatment is bevacizumab. The U.S. FDA, the European Medicines Agency (EMEA), and numerous other regulatory bodies have approved bevacizumab for treating malignancies such as NSCLC at this time [73]. Bevacizumab or ramucirumab added to EGFR TKIs significantly increased PFS in patients with EGFR-mutant NSCLC in recently published large extensive randomized studies [74], In a phase III trial, the inclusion of bevacizumab significantly increased the PFS endpoint from 11.2 months when erlotinib was used alone to 17.9 months when it was used in combination therapy [75].

**Table 3:** Summary of other FDA-approved drugs targeting NSCLC.

Targeted Genes	Drug	Objective Response Rate (ORR)	Median PFS (Months)	Side Effects	Ref.
ROS1	Crizotinib	72.4%	19.2	Visual impairment/nausea/edema/	[35,76]
	Ciritinib	62% (67%) *	19.3	diarrhea/nausea/anorexia/	[43]
	Lorlatinib	41% (62%) *	8.5	dyslipidemia	[43]
	Entrectinib	77%	15.7	weight increase/neutropenia	[77,78]
BRAF	Dabrafenib and trametinib	64% (68%) *	10.8	Fatigue/pyrexia/nausea	[79]
MET	Tepotinib	46%	8.5	Peripheral edema/amylase increased/nausea	[80]
	Capmatinib	41% (68%)	5.4	peripheral edema/Nausea	[81]
RET	Selpercatinib	64% (85%)	18.4	Dry mouth/diarrhea/hypertension	[82,83]
	Pralsetinib	61% (73%) *	16.5 (13) *	anemia/hypertension/neutropenia/	[83]
KRAS	Sotorasib	32%	6.3	diarrhea/nausea/elevated LFT/fatigue	[71]
NTRK	Larotrectinib	75%	35.4	myalgia/hypersensitivity/weight increase	[84]
	Entrectinib	70%	NR	taste disorder/ constipation/fatigue	[84]
HER2	T-DM1	55%	5	Infusion reactions/ thrombocytopenia	[85,86]
	T-DXd	62%	14	nausea /alopecia/anemia	[87]

Abbreviations: ORR, overall response rate; NR, not reported; data outside parentheses are for patients previously treated with platinum-based drugs; data in parentheses with * are for patients who have not previously received systemic therapy.

## 3. Resistance Mechanisms

### 3.1. Mechanisms of Resistance to EGFR TKIs

**Figure 2:**
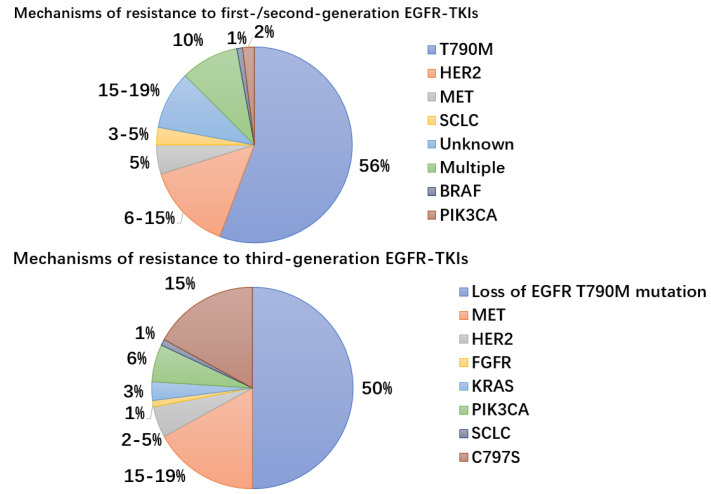
Mechanisms and frequency of resistance to EGFR-TKIs. MET gene amplification, PIK3CA gene mutations, bypass pathway activation, downstream pathway activation, EGFR modification mutations or amplification, and development of small cell lung cancer are examples of resistance mechanisms to EGFR-TKIs (SCLC). Third-generation EGFR-TKIs used as first-line therapy result in C797S mutations.

T790M mutation in exon 20 is present in 50–60% of individuals who are resistant to first-generation EGFR TKIs such as erlotinib [88]. The EGFR protein’s ATP-binding pocket contains the T790 residue, which increases the protein’s affinity for ATP and mediates TKI resistance. T790M decreases Km [ATP], the amount of ATP required to reach a half-maximal response rate when it co-occurs with activating mutations. The effectiveness of first- and second-generation EGFR TKIs is decreased as a result of these biochemical alterations, which restore ATP affinity to a level that is more similar to wild-type EGFR [89]. However, the concurrent decline in k_cat_ results in a reduction in ATP throughput and an increase in enzymatic turnover. This likely explains why the T790M mutation confers a growth disadvantage in cells with classical EGFR-activating mutations in the absence of EGFR TKIs [90]. The majority of the secondary mutations that are not T790M are D761Y, L747S, and T854A. They lessen the sensitivity of mutant EGFR to EGFR-TKIs; however, it is yet unclear how they overcome resistance. One possibility would be that these secondary resistance mutations alter how EGFR is configured and how it interacts with TKIs [8]. Other mechanisms of resistance include MET gene amplification, including EGFR amplification and PIK3CA gene mutations, and conversion to SCLC [91] (Figure 2). Small cell lung cancer (SCLC) can histologically convert into NSCLC in up to 14% of instances, and this transformation is typically accompanied by resistance to the original TKIs [92].

In a recent retrospective analysis of the FLAURA trial, the C797S mutation in EGFR exon 20, which occurs at a frequency of 7% when axitinib was used as first-line therapy and accounts for 10–26% of cases of resistance to second-line axitinib therapy, was examined for the mechanisms of acquired resistance to first-line osimertinib in advanced NSCLC with EGFR mutations [93]. The osimertinib-EGFR covalent link is broken by the EGFR (C797S) mutation, which occurs when the cysteine at codon 797 within the ATP binding site is changed to a serine [94]. By inhibiting their binding to the EGFR active site, the C797S mutation also imparts cross-resistance to other irreversible third-generation TKIs, such as roxitinib, omutinib, and nizatinib [95,96]. MET amplification (15–19%), PIK3CA (6–7%), KRAS (3%), and HER2 amplification (2–5%) were the mechanisms most often found to have acquired resistance [94,97]. Bypass pathway activation, which results in oxitinib resistance through sustained activation of signaling pathways downstream of EGFR, including those mediated by MAPK, STAT, and PI3K-Akt, is most frequently caused by MET gene amplification, which is unrelated to EGFR activation and signaling [98,99]. Recently published early trials for the combination of MET inhibitors with osimertinib showed encouraging outcomes when resistance developed [100].

### 3.2. Mechanisms of Resistance to ALK TKIs

There are three different types of ALK gene mutations: point mutation, amplification, and rearrangement (ALK-R/ALK-A) [101]. The majority of ALK gene mutations take the form of a translocation to another partner gene, creating an overexpressed fusion oncogene in cancer [101]. The oncogenic mechanism of ALK-A in the NB cell line was discovered for the first time in 2002. ALK-A has been shown to cause constitutive activation, which only activates the docking protein SHcC, a member of the Shc family of protein adaptors when it is near ALK receptor substrates [102]. A series of findings in patients with acquired crizotinib resistance were described in which mutations in the ALK TK domain were found in 4 (22%) of 18 patients biopsied after the recurrence of the first-generation crizotinib, including three new mutations and one amplification of the ALK fusion gene [103]. The acquired resistance point mutations identified by ALK included G1269A, C1156Y, I1171T/N/S, S1206C, E1210K, L1152P/R, V11180L, G1128A, F1174V, and L1196M [104,105,106,107]. Patients with crizoltinib-resistant circulating tumor cells (CTC) had repeated mutations in the RTK-KRAS (EGFR, KRAS, BRAF genes), TP53, and other genes in the ALK-independent pathway, according to single CTC sequencing [108]. The activation of bypass signaling pathways such as the activation of transcription co-regulator YAP, EGFR signaling, KIT amplification, the insulin-like growth factor-1 receptor (IGF-1R) pathway, MAPK amplification, BRAF (V600E) mutation, and MET amplification is another component of the resistance mechanism of ALK-TKIs [103,109,110,111,112,113]. MET amplification was present in 15% of tumor samples from patients who were relapsing on next-generation ALK inhibitors, compared to 12% and 22% of tumor biopsies from patients who were progressing on second-generation inhibitors or lorlatinib, respectively. Alteration of MET is a prevalent functional resistance mechanism in lung cancer that is ALK-positive [114].

Numerous studies have demonstrated that the second-generation medicines alectinib, ceritinib, brigatinib, and ensatinib can be more effective than chemotherapy when first-generation ALK inhibitors failed to treat NSCLC patients [115,116,117,118]. In patients treated with second-generation ALK inhibitors, the G1202R mutation is the most prevalent secondary resistant ALK mutation, appearing in 21%, 29%, and 43% of patients treated with ceritinib, alectinib, and brigatinib, respectively [119]. A mid-term review of outcomes in previously untreated patients with advanced ALK-positive NSCLC found that lorlatinib, a third-generation inhibitor of ALK, had significantly longer PFS and more significant proportion of intracranial responses [120]. According to a study, gilteritinib, a TKI approved for the treatment of acute myeloid leukemia (AML) that has relapsed or become resistant to treatment, suppresses both single ALK-TKI-resistant mutants and compound mutants with the mutation I1171N both in vitro and in vivo [121].

### 3.3. Mechanisms of Resistance to ROS1 Inhibitors

Point mutations in the ROS1 kinase domain that render ROS1 fusion-positive cancers resistant to ROS1 TKIs have been identified through studies in both preclinical and clinical settings [122,123]. Point mutations in the ROS1 kinase domain, such as D2033N, G2032 series, L2026M, L2155S series, and S1986F/Y, can cause acquired resistance to crizotinib [35,124]. This mutation reduces the potency of kinase inhibition [42,123,125]. In one study, 55 people’s post-crizotinib and post-lorlatinib biopsies were examined. In 42 post-crizotinib biopsies and 28 post-lorlatinib biopsies, respectively, that were analyzed at various timepoints, ROS1 mutations were discovered in 38% and 46% of the samples. Nearly one-third of patients had the most common mutation, ROS1(G2032R). Post-crizotinib, there were additional ROS1 mutations such as D2033N (2.4%) and S1986F (2.4%) as well as L2086F (3.6%), G2032R/L2086F (3.6%), G2032R/S1986F/L2086F (3.6%). In addition, the increased point mutation with lorlatinib was S1986F/L2000V (3.6%) [124]. Due to the D2033N mutation, which causes the kinase hinge region of ROS1 to change from aspartic acid to asparagine, crizotinib demonstrates significant in vitro drug resistance (inside the ATP binding site) [126,127]. The ROS1 kinase domain mutation L2026M is similar to G2032R in that it results in resistance to crizotinib by altering the gatekeeper position of the binding pocket for the ROS1 inhibitor [123,128]. At codon 2032 in the structural domain of ROS1 kinase, glycine is changed to arginine. This mutation gives resistance to ROS1 kinase inhibition by interfering with drug binding through a spatial site block, while not being present at the gatekeeper residue [129]. Furthermore, the substitution S1986F/Y in the kinase domain blocks important activation sites, increasing kinase activity. L2155S is expected to impart crizotinib resistance through protein failure [130].

Through mutations and/or copy number increases, other RTKs or downstream MAPK pathway effectors are implicated in ROS1-extrinsic resistance mechanisms, showing MAPK system reactivation as a convergent mechanism of resistance [131]. KRAS, NRAS, EGFR, HER2, MET, KIT, BRAF, and MEK are mediators involved in this pathway either as downstream or bypass mediators [122,123,125,127]. KIT and catenin mutations, as well as HER2-mediated bypass signaling, were found to be non-ROS1-dominant resistance pathways in the ROS1 cohort [123]. The study suggests that the unused bypass signaling pathway SHP2 is also associated with the development of drug resistance [123]. Data also suggested that activation of RAS family members can confer resistance to ROS1 inhibitors [132]. KRAS(G12D) and BRAF(V600E^)^ mutations have been linked to crizotinib treatment in the clinical setting, whereas NRAS^Q61K^ has been linked to entrectinib treatment [133].

### 3.4. Mechanisms of Resistance to BRAF Inhibitors

Approximately 50% of BRAF mutations are BRAF(V600E) [47]. Other typical BRAF mutations are BRAF(D594G) and BRAF(G469A/V) mutations, which are present in 35% and 6%, respectively, of BRAF mutant NSCLC patients [134]. The V600E mutation greatly increases the kinase activity of BRAF independent of Ras by stabilizing the active conformation of BRAF by establishing a salt bridge with K507 [135]. BRAF(V600E) mutation causes constitutive BRAF activation in its monomeric form, which promotes MEK-ERK signaling downstream [136]. Although the BRAF(V600) gene-targeting drugs vemurafenib and dabrafenib are clinically effective as monotherapies [137,138], the addition of MEK inhibitors dramatically improves results. The combination of BRAFi and MEKi was superior to the single agent, increasing the ORR rate to 67% and the median PFS to 10.2 months [49]. There is also a therapeutic need for BRAF inhibitors that are effective against non-BRAF(V600E) mutations, which are present in about 50% of BRAF-mutated NSCLC cancers. Increased EGFR signaling through autocrine activation caused by BRAF-independent c-Jun signaling or loss of full-length BRAF (V600E) consistent with the expression of a truncated form of the mutant protein has been the mechanisms of acquired resistance in NSCLC cell lines that were sequentially treated with vemurafenib for BRAF(V600E) mutations [139]. Notably, it has been shown that second-generation BRAF inhibitors (BRAFi) or a combination of BRAF and MEK inhibition can prevent resistance brought on by the production of BRAF(V600E) splice variants (e.g., PLX8394) [139,140]. CRAF kinase expression was one of the resistance mechanisms discovered by Montagut et al. According to this study, mutant cells with high amounts of the CRAF protein may have reduced drug bioavailability [141]. Furthermore, according to these authors, a subpopulation of BRAF-mutant cancer cells may develop that is resistant to the primary inhibitor elevated levels of CRAF protein [141]. A study has shown for the first time how the loss of PTEN results in intrinsic BRAF inhibitor resistance by inhibiting BIM-mediated apoptosis [142].

**Figure 3:**
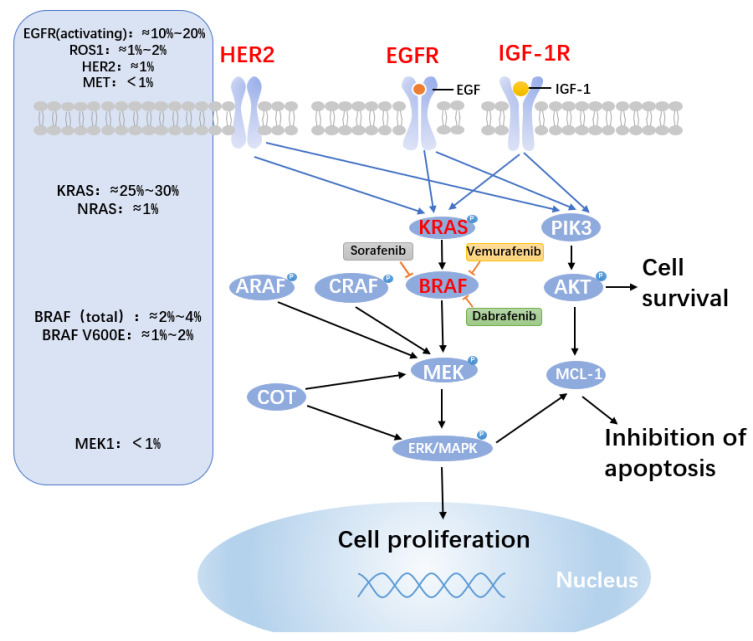
BRAF medication resistance mechanisms and mutation probability. BRAF mutations resulted in altered mitogen-activated protein kinase (MAPK) molecules. The approximate frequencies of frequent driver mutations discovered in the MAPK pathway in lung cancer are shown on the left side of the figure. BRAF valence at codon 600 (V600E) mutations, which cause native activation of BRAF, is only found in 1–2% of lung cancers. In patients with activating BRAF mutations, clinical study of BRAF alone or in combination with downstream MEK inhibition is continuing. On the right, prominent BRAF inhibitors are described. BRAF inhibitor resistance is conferred through cRAF, ARAF, the MAP kinase family member COT, and the pro-survival members of the BCL-2 family MCL-1. Despite BRAF inhibition, increased production of the alternative RAF isoforms (ARAP and CRAF) and MAP3K8/COT can still activate the MAPK pathway. The PI3K and MAPK pathways, which may offer paths around BRAF inhibition and apoptosis, also activate MCL-1.

Unfortunately, the majority of NSCLC patients will experience disease progression within a year of starting BRAF and MEK inhibition as a treatment strategy. According to preclinical and clinical data, in addition to BRAF mutations, resistance mechanisms include the activation of bypass pathways, including the PI3/AKT/mTOR, and the restoration of MAPK signaling that has become ineffective for suppression due to upstream or downstream changes [143] (Figure 3). Mechanisms by which reactivation of MAPK pathway signaling mediates acquired BRAFi resistance have begun to emerge [144]. According to reports, the overexpression or upregulation of RTKs such as PDGFR and EGRF was the first modification that led to RAF inhibitor resistance. Since these modifications stimulate RAS and activate CRAF-MEK-ERK signaling, the proliferation of cancer cells is not dependent on BRAF(V600E) [145,146,147]. RAS mutations, which function similarly to RTK alterations, were identified as the second factor downstream of RTKs contributing to RAF inhibitor resistance [145,146,147,148]. As soon as cancer cells have a large amount of active Ras, the drug-loaded BRAF(V600E) will dimerize with CRAF and activate its catalytic activity [149,150], which has been referred to as the paradoxical effect of RAF inhibitors [151]. As a result, work is still being carried out on the next iteration of BRAFi.

## 4. Discussion

Since lung cancer continues to have the highest mortality rate worldwide, researchers have focused a substantial amount of attention on it. Lung cancer is also a cancer for which targeted therapy development and marketing are most prevalent. In this article, we summarized the eight main NSCLC targeted loci as well as the resistance mechanisms that have been identified. These factors could be important in the future development of targeted treatments. In addition to the targeted loci described in this article, the FDA has also approved other loci such as NTRK and HER2 (Table 3). For patients with advanced NSCLC, anti-PD1/PD-L1 immunotherapy has become a standard treatment option in addition to targeted drugs over the past 10 years. Patients are often chosen based on the tumor mutation burden and/or PD-L1 expression in tumor cells. Mutations in oncogenic factors including EGFR, ALK, BRAF, or MET, which can change the immunological tumor micro-environment, can enhance tolerance to PD1/PD-L1 [152]. Additionally, various cancer patients may develop medication resistance through multiple pathways. To customize targeted therapy for each patient, it is crucial to evaluate their specific resistance mechanisms at the molecular level. Repeating tissue samples is one method for tracking the genetic evolution of therapeutic effects. However, this method is highly invasive, necessitates a high level of patient cooperation, and may be complicated by intra-tumor heterogeneity [153]. In conclusion, individualized medicine has begun to provide substantial benefits for patients with oncogene-driven NSCLC [144], but the treatment of this notorious malignancy still has a long way to go.

## Figures and Tables

**Table 2:** Summary of FDA-approved ALK-TKIs.

Generation	Drug	Objective Response Rate (ORR)	Median PFS (Months)	Side Effects	Ref.
1st	Crizotinib	74%	10.9	Vision disorder/ nausea/diarrhea	[31]
2nd	Ceritinib	73%	16.6	Diarrhea/nausea vomiting	[32,33]
	Alectinib	83%	25.7	AST elevation/CK elevation/fatigue	[32,33]
	Brigatinib	74%	24	nausea/diarrhea/cough	[32,33]
3rd	Lorlatinib	76%	NR	Hypercholesterolemia/edema/peripheral neuropathy/	[33]
	Ensartinib	75%	25.8	rash/ALT elevation/AST elevation	[33,34]

## Data Availability

Not applicable.

## References

[1] Siegel R.L., Miller K.D., Fuchs H.E., Jemal A. (2022). Cancer statistics, 2022. CA Cancer J. Clin..

[2] Alexander M., Kim S.Y., Cheng H. (2020). Update 2020: Management of Non-Small Cell Lung Cancer. Lung.

[3] Broderick S.R. (2020). Adjuvant and Neoadjuvant Immunotherapy in Non-small Cell Lung Cancer. Thorac. Surg. Clin..

[4] La Montagna M., Ginn L., Garofalo M. (2020). Mechanisms of drug resistance mediated by long non-coding RNAs in non-small-cell lung cancer. Cancer Gene Ther..

[5] Imyanitov E.N., Iyevleva A.G., Levchenko E.V. (2021). Molecular testing and targeted therapy for non-small cell lung cancer: Current status and perspectives. Crit. Rev. Oncol..

[6] Rotow J., Bivona T.G. (2017). Understanding and targeting resistance mechanisms in NSCLC. Nat. Rev. Cancer.

[7] Gazdar A.F. (2009). Activating and resistance mutations of EGFR in non-small-cell lung cancer: Role in clinical response to EGFR tyrosine kinase inhibitors. Oncogene.

[8] Huang L., Fu L. (2015). Mechanisms of resistance to EGFR tyrosine kinase inhibitors. Acta Pharm. Sin. B.

[9] Meador C.B., Hata A.N. (2020). Acquired resistance to targeted therapies in NSCLC: Updates and evolving insights. Pharmacol. Ther..

[10] Liu Q., Yu S., Zhao W., Qin S., Chu Q., Wu K. (2018). EGFR-TKIs resistance via EGFR-independent signaling pathways. Mol. Cancer.

[11] da Cunha Santos G., Shepherd F.A., Tsao M.S. (2011). EGFR mutations and lung cancer. Annu. Rev. Pathol..

[12] Rosell R., Carcereny E., Gervais R., Vergnenegre A., Massuti B., Felip E., Palmero R., Garcia-Gomez R., Pallares C., Sanchez J.M. (2012). Erlotinib versus standard chemotherapy as first-line treatment for European patients with advanced EGFR mutation-positive non-small-cell lung cancer (EURTAC): A multicentre, open-label, randomised phase 3 trial. Lancet Oncol..

[13] Westover D., Zugazagoitia J., Cho B.C., Lovly C.M., Paz-Ares L. (2018). Mechanisms of acquired resistance to first- and second-generation EGFR tyrosine kinase inhibitors. Ann. Oncol..

[14] Maemondo M., Inoue A., Kobayashi K., Sugawara S., Oizumi S., Isobe H., Gemma A., Harada M., Yoshizawa H., Kinoshita I. (2010). Gefitinib or chemotherapy for non-small-cell lung cancer with mutated EGFR. N. Engl. J. Med..

[15] Sequist L.V., Yang J.C.-H., Yamamoto N., Obyrne K., Hirsh V., Mok T., Geater S.L., Orlov S., Tsai C.-M., Boyer M. (2013). Phase III study of afatinib or cisplatin plus pemetrexed in patients with metastatic lung adenocarcinoma with EGFR mutations. J. Clin. Oncol..

[16] Remon J., Steuer C., Ramalingam S., Felip E. (2018). Osimertinib and other third-generation EGFR TKI in EGFR-mutant NSCLC patients. Ann. Oncol..

[17] Soria J.C., Ohe Y., Vansteenkiste J., Reungwetwattana T., Chewaskulyong B., Lee K.H., Dechaphunkul A., Imamura F., Nogami N., Kurata T. (2018). Osimertinib in Untreated EGFR-Mutated Advanced Non-Small-Cell Lung Cancer. N. Engl. J. Med..

[18] Liang J.L., Ren X.C., Lin Q. (2014). Treating advanced non-small-cell lung cancer in Chinese patients: Focus on icotinib. Onco. Targets Ther..

[19] Juan O., Popat S. (2017). Treatment choice in epidermal growth factor receptor mutation-positive non-small cell lung carcinoma: Latest evidence and clinical implications. Ther. Adv. Med. Oncol..

[20] Lavacchi D., Mazzoni F., Giaccone G. (2019). Clinical evaluation of dacomitinib for the treatment of metastatic non-small cell lung cancer (NSCLC): Current perspectives. Drug Des. Devel. Ther..

[21] Le T., Gerber D.E. (2019). Newer-Generation EGFR Inhibitors in Lung Cancer: How Are They Best Used?. Cancers.

[22] Kohno T., Nakaoku T., Tsuta K., Tsuchihara K., Matsumoto S., Yoh K., Goto K. (2015). Beyond ALK-RET, ROS1 and other oncogene fusions in lung cancer. Transl. Lung Cancer Res..

[23] Duyster J., Bai R.-Y., Morris S.W. (2001). Translocations involving anaplastic lymphoma kinase (ALK). Oncogene.

[24] Wu J., Savooji J., Liu D. (2016). Second- and third-generation ALK inhibitors for non-small cell lung cancer. J. Hematol. Oncol..

[25] Zhang S.S., Nagasaka M., Zhu V.W., Ou S.-H.I. (2021). Going beneath the tip of the iceberg. Identifying and understanding EML4-ALK variants and TP53 mutations to optimize treatment of ALK fusion positive (ALK+) NSCLC. Lung Cancer.

[26] Sullivan I., Planchard D. (2015). ALK inhibitors in non-small cell lung cancer: The latest evidence and developments. Ther. Adv. Med. Oncol..

[27] Kwak E.L., Bang Y.-J., Camidge D.R., Shaw A.T., Solomon B., Maki R.G., Ou S.-H.I., Dezube B.J., Jänne P.A., Costa D.B. (2010). Anaplastic lymphoma kinase inhibition in non-small-cell lung cancer. N. Engl. J. Med..

[28] Markham A. (2017). Brigatinib: First Global Approval. Drugs.

[29] Sakamoto H., Tsukaguchi T., Hiroshima S., Kodama T., Kobayashi T., Fukami T.A., Oikawa N., Tsukuda T., Ishii N., Aoki Y. (2011). CH5424802, a selective ALK inhibitor capable of blocking the resistant gatekeeper mutant. Cancer Cell.

[30] Karachaliou N., Santarpia M., Cao M.G., Teixido C., Sosa A.E., Berenguer J., Capote A.R., Altavilla G., Rosell R. (2017). Anaplastic lymphoma kinase inhibitors in phase I and phase II clinical trials for non-small cell lung cancer. Expert Opin. Investig. Drugs.

[31] Prabhash K., Noronha V., Joshi A., Desai S., Sahu A. (2013). Crizotinib: A comprehensive review. South Asian J. Cancer.

[32] Xia B., Nagasaka M., Zhu V.W., Ou S.-H.I., Soo R.A. (2020). How to select the best upfront therapy for metastatic disease? Focus on ALK-rearranged non-small cell lung cancer (NSCLC). Transl. Lung Cancer Res..

[33] Wang Y., Yuan X., Xiong J., Hao Z., Peng X., Chen W., Cui L., Li H., Wang X., He X. (2020). Pharmacology and Clinical Evaluation of Ensartinib Hydrochloride Capsule. Zhongguo Fei Ai Za Zhi.

[34] Horn L., Wang Z., Wu G., Poddubskaya E., Mok T., Reck M., Wakelee H., Chiappori A.A., Lee D.H., Breder V. (2021). Ensartinib vs. Crizotinib for Patients With Anaplastic Lymphoma Kinase-Positive Non-Small Cell Lung Cancer: A Randomized Clinical Trial. JAMA Oncol..

[35] D’Angelo A., Sobhani N., Chapman R., Bagby S., Bortoletti C., Traversini M., Ferrari K., Voltolini L., Darlow J., Roviello G. (2020). Focus on ROS1-Positive Non-Small Cell Lung Cancer (NSCLC): Crizotinib, Resistance Mechanisms and the Newer Generation of Targeted Therapies. Cancers.

[36] Acquaviva J., Wong R., Charest A. (2009). The multifaceted roles of the receptor tyrosine kinase ROS in development and cancer. Biochim. Biophys. Acta.

[37] Bergethon K., Shaw A.T., Ou S.-H.I., Katayama R., Lovly C.M., McDonald N.T., Massion P.P., Siwak-Tapp C., Gonzalez A., Fang R. (2012). ROS1 rearrangements define a unique molecular class of lung cancers. J. Clin. Oncol..

[38] Guaitoli G., Bertolini F., Bettelli S., Manfredini S., Maur M., Trudu L., Aramini B., Masciale V., Grisendi G., Dominici M. (2021). Deepening the Knowledge of ROS1 Rearrangements in Non-Small Cell Lung Cancer: Diagnosis, Treatment, Resistance and Concomitant Alterations. Int. J. Mol. Sci..

[39] Patil T., Simons E., Mushtaq R., Pacheco J., Doebele R., Bowles D. (2019). Targeted therapies for ROS1-rearranged non-small cell lung cancer. Drugs Today.

[40] De Giglio A., Lamberti G., Facchinetti F., Genova C., Andrini E., Bello M.G.D., Tiseo M., Metro G., Chiari R., Ricciuti B. (2020). Treatment Patterns and Clinical Outcomes Among Patients With ROS1-rearranged Non-small-cell Lung Cancer Progressing on Crizotinib. Clin. Lung Cancer.

[41] Lin J.J., Shaw A.T. (2017). Recent Advances in Targeting ROS1 in Lung Cancer. J. Thorac. Oncol..

[42] Dziadziuszko R., Le A.T., Wrona A., Jassem J., Camidge D.R., Varella-Garcia M., Aisner D.L., Doebele R.C. (2016). An Activating KIT Mutation Induces Crizotinib Resistance in ROS1-Positive Lung Cancer. J. Thorac. Oncol..

[43] Azelby C.M., Sakamoto M.R., Bowles D.W. (2021). ROS1 Targeted Therapies: Current Status. Curr. Oncol. Rep..

[44] Negrao M.V., Raymond V.M., Lanman R.B., Robichaux J.P., He J., Nilsson M.B., Ng P.K., Amador B.E., Roarty E.B., Nagy R.J. (2020). Molecular Landscape of BRAF-Mutant NSCLC Reveals an Association Between Clonality and Driver Mutations and Identifies Targetable Non-V600 Driver Mutations. J. Thorac. Oncol..

[45] Loo E., Khalili P., Beuhler K., Siddiqi I., Vasef M.A. (2018). BRAF V600E Mutation Across Multiple Tumor Types: Correlation Between DNA-based Sequencing and Mutation-specific Immunohistochemistry. Appl. Immunohistochem. Mol. Morphol..

[46] Ikenoue T., Hikiba Y., Kanai F., Aragaki J., Tanaka Y., Imamura J., Imamura T., Ohta M., Ijichi H., Tateishi K. (2004). Different effects of point mutations within the B-Raf glycine-rich loop in colorectal tumors on mitogen-activated protein/extracellular signal-regulated kinase kinase/extracellular signal-regulated kinase and nuclear factor kappaB pathway and cellular transformation. Cancer Res..

[47] Marchetti A., Felicioni L., Malatesta S., Sciarrotta M.G., Guetti L., Chella A., Viola P., Pullara C., Mucilli F., Buttitta F. (2011). Clinical features and outcome of patients with non-small-cell lung cancer harboring BRAF mutations. J. Clin. Oncol..

[48] Odogwu L., Mathieu L., Blumenthal G., Larkins E., Goldberg K.B., Griffin N., Bijwaard K., Lee E.Y., Philip R., Jiang X. (2018). FDA Approval Summary: Dabrafenib and Trametinib for the Treatment of Metastatic Non-Small Cell Lung Cancers Harboring BRAF V600E Mutations. Oncologist.

[49] O’Leary C.G., Andelkovic V., Ladwa R., Pavlakis N., Zhou C., Hirsch F., Richard D., O’Byrne K. (2019). Targeting BRAF mutations in non-small cell lung cancer. Transl. Lung Cancer Res..

[50] Mazieres J., Cropet C., Montané L., Barlesi F., Souquet P.S., Quantin X., Dubos-Arvis C., Otto J., Favier L., Avrillon V. (2020). Vemurafenib in non-small-cell lung cancer patients with BRAF(V600) and BRAF(nonV600) mutations. Ann. Oncol..

[51] Schreck K.C., Grossman S.A., Pratilas C.A. (2019). BRAF Mutations and the Utility of RAF and MEK Inhibitors in Primary Brain Tumors. Cancers.

[52] Park M., Dean M., Cooper C.S., Schmidt M., O’Brien S.J., Blair D.G., Woude G.F.V. (1986). Mechanism of met oncogene activation. Cell.

[53] Santarpia M., Massafra M., Gebbia V., D’Aquino A., Garipoli C., Altavilla G., Rosell R. (2021). A narrative review of MET inhibitors in non-small cell lung cancer with MET exon 14 skipping mutations. Transl. Lung Cancer Res..

[54] Skead G., Govender D. (2015). Gene of the month: MET. J. Clin. Pathol..

[55] Drilon A., Cappuzzo F., Ou S.-H.I., Camidge D.R. (2017). Targeting MET in Lung Cancer: Will Expectations Finally Be MET?. J. Thorac. Oncol..

[56] Ma P.C., Kijima T., Maulik G., Fox E.A., Sattler M., Griffin J.D., Johnson B.E., Salgia R. (2003). c-MET mutational analysis in small cell lung cancer: Novel juxtamembrane domain mutations regulating cytoskeletal functions. Cancer Res..

[57] Awad M.M., Oxnard G.R., Jackman D.M., Savukoski D.O., Hall D., Shivdasani P., Heng J.C., Dahlberg S.E., Jänne P.A., Verma S. (2016). MET Exon 14 Mutations in Non-Small-Cell Lung Cancer Are Associated With Advanced Age and Stage-Dependent MET Genomic Amplification and c-Met Overexpression. J. Clin. Oncol..

[58] Han S., Ma X., Fang J. (2020). Progress on Mechanism of MET Gene Mutation and Targeted Drugs in Non-small Cell Lung Cancer. Zhongguo Fei Ai Za Zhi.

[59] Fujino T., Suda K., Mitsudomi T. (2020). Emerging MET tyrosine kinase inhibitors for the treatment of non-small cell lung cancer. Expert Opin. Emerg. Drugs.

[60] Mathieu L.N., Larkins E., Akinboro O., Roy P., Amatya A.K., Fiero M.H., Mishra-Kalyani P.S., Helms W.S., Myers C.E., Skinner A.M. (2022). FDA Approval Summary: Capmatinib and Tepotinib for the Treatment of Metastatic NSCLC Harboring MET Exon 14 Skipping Mutations or Alterations. Clin. Cancer Res..

[61] Drilon A., Hu Z.I., Lai G.G.Y., Tan D.S.W. (2017). Targeting RET-driven cancers: Lessons from evolving preclinical and clinical landscapes. Nat. Rev. Clin. Oncol..

[62] Bronte G., Ulivi P., Verlicchi A., Cravero P., Delmonte A., Crinò L. (2019). Targeting RET-rearranged non-small-cell lung cancer: Future prospects. Lung Cancer.

[63] Ferrara R., Auger N., Auclin E., Besse B. (2018). Clinical and Translational Implications of RET Rearrangements in Non-Small Cell Lung Cancer. J. Thorac. Oncol..

[64] Wright K.M. (2020). FDA Approves Pralsetinib for Treatment of Adults With Metastatic RET Fusion-Positive NSCLC. Oncology.

[65] Nguyen L., Monestime S. (2022). Pralsetinib: Treatment of metastatic RET fusion-positive non-small cell lung cancer. Am. J. Health Syst. Pharm..

[66] Liu P., Wang Y., Li X. (2019). Targeting the untargetable KRAS in cancer therapy. Acta Pharm. Sin. B.

[67] Reck M., Carbone D., Garassino M., Barlesi F. (2021). Targeting KRAS in non-small-cell lung cancer: Recent progress and new approaches. Ann. Oncol..

[68] Canon J., Rex K., Saiki A.Y., Mohr C., Cooke K., Bagal D., Gaida K., Holt T., Knutson C.G., Koppada N. (2019). The clinical KRAS(G12C) inhibitor AMG 510 drives anti-tumour immunity. Nature.

[69] Blair H.A. (2021). Sotorasib: First Approval. Drugs.

[70] Skoulidis F., Li B.T., Dy G.K., Price T.J., Falchook G.S., Wolf J., Italiano A., Schuler M., Borghaei H., Barlesi F. (2021). Sotorasib for Lung Cancers with KRAS p.G12C Mutation. N. Engl. J. Med..

[71] Hong D.S., Fakih M.G., Strickler J.H., Desai J., Durm G.A., Shapiro G.I., Falchook G.S., Price T.J., Sacher A., Denlinger C.S. (2020). KRAS(G12C) Inhibition with Sotorasib in Advanced Solid Tumors. N. Engl. J. Med..

[72] Nakajima E.C., Drezner N., Li X., Mishra-Kalyani P.S., Liu Y., Zhao H., Bi Y., Liu J., Rahman A., Wearne E. (2021). FDA Approval Summary: Sotorasib for KRAS G12C-Mutated Metastatic NSCLC. Clin. Cancer Res..

[73] Melincovici C.S., Boşca A.B., Şuşman S., Mărginean M., Mihu C., Istrate M., Moldovan I.M., Roman A.L., Mihu C.M. (2018). Vascular endothelial growth factor (VEGF)—Key factor in normal and pathological angiogenesis. Rom. J. Morphol. Embryol..

[74] Le X., Nilsson M., Goldman J., Reck M., Nakagawa K., Kato T., Ares L.P., Frimodt-Moller B., Wolff K., Visseren-Grul C. (2021). Dual EGFR-VEGF Pathway Inhibition: A Promising Strategy for Patients With EGFR-Mutant NSCLC. J. Thorac. Oncol..

[75] Hafner S. (2021). First-line anti-VEGF plus EGFR-TKI in EGFR-mutant NSCLC: Adding the ARTEMIS trial to the puzzle of current evidence. Signal Transduct. Target. Ther..

[76] Shaw A., Riely G., Bang Y.-J., Kim D.-W., Camidge D., Solomon B., Varella-Garcia M., Iafrate A., Shapiro G., Usari T. (2019). Crizotinib in ROS1-rearranged advanced non-small-cell lung cancer (NSCLC): Updated results, including overall survival, from PROFILE 1001. Ann. Oncol..

[77] Drilon A., Siena S., Dziadziuszko R., Barlesi F., Krebs M.G., Shaw A.T., de Braud F., Rolfo C., Ahn M.-J., Wolf J. (2019). Entrectinib in ROS1 fusion-positive non-small-cell lung cancer: Integrated analysis of three phase 1–2 trials. Lancet Oncol..

[78] Dziadziuszko R., Krebs M.G., De Braud F., Siena S., Drilon A., Doebele R.C., Patel M.R., Cho B.C., Liu S.V., Ahn M.-J. (2021). Updated Integrated Analysis of the Efficacy and Safety of Entrectinib in Locally Advanced or Metastatic ROS1 Fusion-Positive Non-Small-Cell Lung Cancer. J. Clin. Oncol..

[79] Auliac J.-B., Bayle S., Do P., Le Garff G., Roa M., Falchero L., Huchot E., Quéré G., Jeannin G., Métivier A.-C. (2020). Efficacy of Dabrafenib Plus Trametinib Combination in Patients with BRAF V600E-Mutant NSCLC in Real-World Setting: GFPC 01-2019. Cancers.

[80] Paik P.K., Felip E., Veillon R., Sakai H., Cortot A.B., Garassino M.C., Mazieres J., Viteri S., Senellart H., Van Meerbeeck J. (2020). Tepotinib in Non-Small-Cell Lung Cancer with MET Exon 14 Skipping Mutations. N. Engl. J. Med..

[81] Wu Y.-L., Smit E.F., Bauer T.M. (2021). Capmatinib for patients with non-small cell lung cancer with MET exon 14 skipping mutations: A review of preclinical and clinical studies. Cancer Treat. Rev..

[82] Drilon A., Oxnard G.R., Tan D.S.W., Loong H.H.F., Johnson M., Gainor J., McCoach C.E., Gautschi O., Besse B., Cho B.C. (2020). Efficacy of Selpercatinib in RET Fusion-Positive Non-Small-Cell Lung Cancer. N. Engl. J. Med..

[83] Cascetta P., Sforza V., Manzo A., Carillio G., Palumbo G., Esposito G., Montanino A., Costanzo R., Sandomenico C., De Cecio R. (2021). RET Inhibitors in Non-Small-Cell Lung Cancer. Cancers.

[84] Qin H., Patel M.R. (2022). The Challenge and Opportunity of NTRK Inhibitors in Non-Small Cell Lung Cancer. Int. J. Mol. Sci..

[85] Riudavets M., Sullivan I., Abdayem P., Planchard D. (2021). Targeting HER2 in non-small-cell lung cancer (NSCLC): A glimpse of hope? An updated review on therapeutic strategies in NSCLC harbouring HER2 alterations. ESMO Open.

[86] Li B.T., Shen R., Buonocore D., Olah Z.T., Ni A., Ginsberg M.S., Ulaner G.A., Offin M., Feldman D., Hembrough T. (2018). Ado-Trastuzumab Emtansine for Patients With HER2-Mutant Lung Cancers: Results From a Phase II Basket Trial. J. Clin. Oncol..

[87] Azar I., Alkassis S., Fukui J., Alsawah F., Fedak K., Al Hallak M.N., Sukari A., Nagasaka M. (2021). Spotlight on Trastuzumab Deruxtecan (DS-8201,T-DXd) for HER2 Mutation Positive Non-Small Cell Lung Cancer. Lung Cancer.

[88] Camidge D.R., Pao W., Sequist L.V. (2014). Acquired resistance to TKIs in solid tumours: Learning from lung cancer. Nat. Rev. Clin. Oncol..

[89] Yun C.-H., Mengwasser K.E., Toms A.V., Woo M.S., Greulich H., Wong K.K., Meyerson M., Eck M.J. (2008). The T790M mutation in EGFR kinase causes drug resistance by increasing the affinity for ATP. Proc. Natl. Acad. Sci. USA.

[90] Chmielecki J., Foo J., Oxnard G.R., Hutchinson K., Ohashi K., Somwar R., Wang L., Amato K.R., Arcila M., Sos M.L. (2011). Optimization of dosing for EGFR-mutant non-small cell lung cancer with evolutionary cancer modeling. Sci. Transl. Med..

[91] Sequist L.V., Waltman B.A., Dias-Santagata D., Digumarthy S., Turke A.B., Fidias P., Bergethon K., Shaw A.T., Gettinger S., Cosper A.K. (2011). Genotypic and histological evolution of lung cancers acquiring resistance to EGFR inhibitors. Sci. Transl. Med..

[92] Rath B., Plangger A., Hamilton G. (2020). Non-small cell lung cancer-small cell lung cancer transformation as mechanism of resistance to tyrosine kinase inhibitors in lung cancer. Cancer Drug Resist.

[93] Mok T.S., Wu Y.-L., Ahn M.J., Garassino M.C., Kim H.R., Ramalingam S.S., Shepherd F.A., He Y., Akamatsu H., Theelen W.S.M.E. (2017). Osimertinib or Platinum-Pemetrexed in EGFR T790M-Positive Lung Cancer. N. Engl. J. Med..

[94] Leonetti A., Sharma S., Minari R., Perego P., Giovannetti E., Tiseo M. (2019). Resistance mechanisms to osimertinib in EGFR-mutated non-small cell lung cancer. Br. J. Cancer.

[95] Yang Z., Yang N., Ou Q., Xiang Y., Jiang T., Wu X., Bao H., Tong X., Wang X., Shao Y.W. (2018). Investigating Novel Resistance Mechanisms to Third-Generation EGFR Tyrosine Kinase Inhibitor Osimertinib in Non-Small Cell Lung Cancer Patients. Clin. Cancer Res..

[96] Niederst M.J., Hu H., Mulvey H.E., Lockerman E.L., Garcia A.R., Piotrowska Z., Sequist L.V., Engelman J.A. (2015). The Allelic Context of the C797S Mutation Acquired upon Treatment with Third-Generation EGFR Inhibitors Impacts Sensitivity to Subsequent Treatment Strategies. Clin. Cancer Res..

[97] Takeda M., Nakagawa K. (2019). First- and Second-Generation EGFR-TKIs Are All Replaced to Osimertinib in Chemo-Naive EGFR Mutation-Positive Non-Small Cell Lung Cancer?. Int. J. Mol. Sci..

[98] Yu H.A., Arcila M.E., Rekhtman N., Sima C.S., Zakowski M.F., Pao W., Kris M.G., Miller V.A., Ladanyi M., Riely G.J. (2013). Analysis of tumor specimens at the time of acquired resistance to EGFR-TKI therapy in 155 patients with EGFR-mutant lung cancers. Clin. Cancer Res..

[99] Chabon J.J., Simmons A.D., Lovejoy A.F., Esfahani M.S., Newman A.M., Haringsma H.J., Kurtz D.M., Stehr H., Scherer F., Karlovich C.A. (2016). Circulating tumour DNA profiling reveals heterogeneity of EGFR inhibitor resistance mechanisms in lung cancer patients. Nat. Commun..

[100] Schmid S., Li J., Leighl N.B. (2020). Mechanisms of osimertinib resistance and emerging treatment options. Lung Cancer.

[101] Du X., Shao Y., Qin H.-F., Tai Y.-H., Gao H.-J. (2018). ALK-rearrangement in non-small-cell lung cancer (NSCLC). Thorac. Cancer.

[102] Miyake I., Hakomori Y., Shinohara A., Gamou T., Saito M., Iwamatsu A., Sakai R. (2002). Activation of anaplastic lymphoma kinase is responsible for hyperphosphorylation of ShcC in neuroblastoma cell lines. Oncogene.

[103] Katayama R., Shaw A.T., Khan T.M., Mino-Kenudson M., Solomon B.J., Halmos B., Jessop N.A., Wain J.C., Yeo A.T., Benes C. (2012). Mechanisms of acquired crizotinib resistance in ALK-rearranged lung Cancers. Sci. Transl. Med..

[104] Kim S., Kim T.M., Kim D.-W., Go H., Keam B., Lee S.-H., Ku J.-L., Chung D.H., Heo D.S. (2013). Heterogeneity of genetic changes associated with acquired crizotinib resistance in ALK-rearranged lung cancer. J. Thorac. Oncol..

[105] Ai X., Niu X., Chang L., Chen R., Ou S.-H.I., Lu S. (2018). Next generation sequencing reveals a novel ALK G1128A mutation resistant to crizotinib in an ALK-Rearranged NSCLC patient. Lung Cancer.

[106] Yanagitani N., Uchibori K., Koike S., Tsukahara M., Kitazono S., Yoshizawa T., Horiike A., Ohyanagi F., Tambo Y., Nishikawa S. (2020). Drug resistance mechanisms in Japanese anaplastic lymphoma kinase-positive non-small cell lung cancer and the clinical responses based on the resistant mechanisms. Cancer Sci..

[107] Dehghanian F., Kay M., Vallian S. (2017). F1174V mutation alters the ALK active conformation in response to Crizotinib in NSCLC: Insight from molecular simulations. J. Mol. Graph. Model..

[108] Pailler E., Faugeroux V., Oulhen M., Mezquita L., Laporte M., Honoré A., Lecluse Y., Queffelec P., Ngo-Camus M., Nicotra C. (2019). Acquired Resistance Mutations to ALK Inhibitors Identified by Single Circulating Tumor Cell Sequencing in ALK-Rearranged Non-Small-Cell Lung Cancer. Clin. Cancer Res..

[109] Sasaki T., Koivunen J., Ogino A., Yanagita M., Nikiforow S., Zheng W., Lathan C., Marcoux J.P., Du J., Okuda K. (2011). A novel ALK secondary mutation and EGFR signaling cause resistance to ALK kinase inhibitors. Cancer Res..

[110] Lovly C.M., McDonald N.T., Chen H., Ortiz-Cuaran S., Heukamp L.C., Yan Y., Florin A., Ozretić L., Lim D., Wang L. (2014). Rationale for co-targeting IGF-1R and ALK in ALK fusion-positive lung cancer. Nat. Med..

[111] Crystal A.S., Shaw A.T., Sequist L.V., Friboulet L., Niederst M.J., Lockerman E.L., Frias R.L., Gainor J.F., Amzallag A., Greninger P. (2014). Patient-derived models of acquired resistance can identify effective drug combinations for cancer. Science.

[112] Tsuji T., Ozasa H., Aoki W., Aburaya S., Funazo T., Furugaki K., Yoshimura Y., Ajimizu H., Okutani R., Yasuda Y. (2019). Alectinib Resistance in ALK-Rearranged Lung Cancer by Dual Salvage Signaling in a Clinically Paired Resistance Model. Mol. Cancer Res..

[113] Shi R., Filho S.N.M., Li M., Fares A., Weiss J., Pham N.-A., Ludkovski O., Raghavan V., Li Q., Ravi D. (2020). BRAF V600E mutation and MET amplification as resistance pathways of the second-generation anaplastic lymphoma kinase (ALK) inhibitor alectinib in lung cancer. Lung Cancer.

[114] Dagogo-Jack I., Yoda S., Lennerz J.K., Langenbucher A., Lin J.J., Rooney M.M., Prutisto-Chang K., Oh A., Adams N.A., Yeap B.Y. (2020). MET Alterations Are a Recurring and Actionable Resistance Mechanism in ALK-Positive Lung Cancer. Clin. Cancer Res..

[115] Novello S., Mazières J., Oh I.-J., de Castro J., Migliorino M., Helland A., Dziadziuszko R., Griesinger F., Kotb A., Zeaiter A. (2018). Alectinib versus chemotherapy in crizotinib-pretreated anaplastic lymphoma kinase (ALK)-positive non-small-cell lung cancer: Results from the phase III ALUR study. Ann. Oncol..

[116] Shaw A.T., Kim T.M., Crinò L., Gridelli C., Kiura K., Liu G., Novello S., Bearz A., Gautschi O., Mok T. (2017). Ceritinib versus chemotherapy in patients with ALK-rearranged non-small-cell lung cancer previously given chemotherapy and crizotinib (ASCEND-5): A randomised, controlled, open-label, phase 3 trial. Lancet Oncol..

[117] Yang Y., Zhou J., Zhou J., Feng J., Zhuang W., Chen J., Zhao J., Zhong W., Zhao Y., Zhang Y. (2019). Efficacy, safety, and biomarker analysis of ensartinib in crizotinib-resistant, ALK-positive non-small-cell lung cancer: A multicentre, phase 2 trial. Lancet Respir. Med..

[118] Camidge D.R., Kim D.-W., Tiseo M., Langer C.J., Ahn M.-J., Shaw A.T., Huber R.M., Hochmair M.J., Lee D.H., Bazhenova L.A. (2018). Exploratory Analysis of Brigatinib Activity in Patients With Anaplastic Lymphoma Kinase-Positive Non-Small-Cell Lung Cancer and Brain Metastases in Two Clinical Trials. J. Clin. Oncol..

[119] Gainor J.F., Dardaei L., Yoda S., Friboulet L., Leshchiner I., Katayama R., Dagogo-Jack I., Gadgeel S., Schultz K., Singh M. (2016). Molecular Mechanisms of Resistance to First- and Second-Generation ALK Inhibitors in ALK-Rearranged Lung Cancer. Cancer Discov..

[120] Shaw A.T., Bauer T.M., de Marinis F., Felip E., Goto Y., Liu G., Mazieres J., Kim D.-W., Mok T., Polli A. (2020). First-Line Lorlatinib or Crizotinib in Advanced ALK-Positive Lung Cancer. N. Engl. J. Med..

[121] Mizuta H., Okada K., Araki M., Adachi J., Takemoto A., Kutkowska J., Maruyama K., Yanagitani N., Oh-Hara T., Watanabe K. (2021). Gilteritinib overcomes lorlatinib resistance in ALK-rearranged cancer. Nat. Commun..

[122] Gainor J.F., Tseng D., Yoda S., Dagogo-Jack I., Friboulet L., Lin J.J., Hubbeling H.G., Dardaei L., Farago A.F., Schultz K.R. (2017). Patterns of Metastatic Spread and Mechanisms of Resistance to Crizotinib in ROS1-Positive Non-Small-Cell Lung Cancer. JCO Precis. Oncol..

[123] McCoach C.E., Le A.T., Aisner D., Gowan K., Jones K.L., Merrick D., Bunn P.A., Purcell W.T., Varella-Garcia M., Camidge D.R. (2016). Resistance Mechanisms to Targeted Therapies in ROS1(+) and ALK(+) Non-small Cell Lung Cancer. J. Clin. Oncol..

[124] Lin J.J., Choudhury N.J., Yoda S., Zhu V.W., Johnson T.W., Sakhtemani R., Dagogo-Jack I., Digumarthy S.R., Lee C., Do A. (2021). Spectrum of Mechanisms of Resistance to Crizotinib and Lorlatinib in ROS1 Fusion-Positive Lung Cancer. Clin. Cancer Res..

[125] Facchinetti F., Loriot Y., Cassin-Kuo M.-S., Mahjoubi L., Lacroix L., Planchard D., Besse B., Farace F., Auger N., Remon J. (2016). Crizotinib-Resistant ROS1 Mutations Reveal a Predictive Kinase Inhibitor Sensitivity Model for ROS1- and ALK-Rearranged Lung Cancers. Clin. Cancer Res..

[126] Facchinetti F., Rossi G., Bria E., Soria J.-C., Besse B., Minari R., Friboulet L., Tiseo M. (2017). Oncogene addiction in non-small cell lung cancer: Focus on ROS1 inhibition. Cancer Treat. Rev..

[127] Drilon A., Somwar R., Wagner J.P., Vellore N.A., Eide C.A., Zabriskie M.S., Arcila M.E., Hechtman J.F., Wang L., Smith R.S. (2016). A Novel Crizotinib-Resistant Solvent-Front Mutation Responsive to Cabozantinib Therapy in a Patient with ROS1-Rearranged Lung Cancer. Clin. Cancer Res..

[128] Zou H.Y., Li Q., Engstrom L.D., West M., Appleman V., Wong K.A., McTigue M., Deng Y.-L., Liu W., Brooun A. (2015). PF-06463922 is a potent and selective next-generation ROS1/ALK inhibitor capable of blocking crizotinib-resistant ROS1 mutations. Proc. Natl. Acad. Sci. USA.

[129] Awad M.M., Katayama R., McTigue M., Liu W., Deng Y.-L., Brooun A., Friboulet L., Huang D., Falk M.D., Timofeevski S. (2013). Acquired resistance to crizotinib from a mutation in CD74-ROS1. N. Engl. J. Med..

[130] Song A., Kim T.M., Kim D.-W., Kim S., Keam B., Lee S.-H., Heo D.S. (2015). Molecular Changes Associated with Acquired Resistance to Crizotinib in ROS1-Rearranged Non-Small Cell Lung Cancer. Clin. Cancer Res..

[131] Drilon A., Jenkins C., Iyer S., Schoenfeld A., Keddy C., Davare M.A. (2021). ROS1-dependent cancers-biology, diagnostics and therapeutics. Nat. Rev. Clin. Oncol..

[132] Cargnelutti M., Corso S., Pergolizzi M., Mévellec L., Aisner D.L., Dziadziuszko R., Varella-Garcia M., Comoglio P.M., Doebele R.C., Vialard J. (2014). Activation of RAS family members confers resistance to ROS1 targeting drugs. Oncotarget.

[133] Zhu Y.-C., Lin X.-P., Li X.-F., Wu L.-X., Chen H.-F., Wang W.-X., Xu C.-W., Shen J.-F., Wei J.-G., Du K.-Q. (2017). Concurrent ROS1 gene rearrangement and KRAS mutation in lung adenocarcinoma: A case report and literature review. Thorac. Cancer.

[134] Litvak A.M., Paik P.K., Woo K.M., Sima C.S., Hellmann M.D., Arcila M.E., Ladanyi M., Rudin C.M., Kris M.G., Riely G.J. (2014). Clinical characteristics and course of 63 patients with BRAF mutant lung cancers. J. Thorac. Oncol..

[135] Degirmenci U., Wang M., Hu J. (2020). Targeting Aberrant RAS/RAF/MEK/ERK Signaling for Cancer Therapy. Cells.

[136] Yao Z., Torres N.M., Torres N.M., Gao Y., Gao Y., Li Q., de Stanchina E., de Stanchina E., Solit D.B., Poulikakos P.I. (2015). BRAF Mutants Evade ERK-Dependent Feedback by Different Mechanisms that Determine Their Sensitivity to Pharmacologic Inhibition. Cancer Cell.

[137] Planchard D., Kim T.M., Mazieres J., Quoix E., Riely G., Barlesi F., Souquet P.-J., Smit E.F., Groen H.J.M., Kelly R.J. (2016). Dabrafenib in patients with BRAF(V600E)-positive advanced non-small-cell lung cancer: A single-arm, multicentre, open-label, phase 2 trial. Lancet Oncol..

[138] Hyman D.M., Puzanov I., Subbiah V., Faris J.E., Chau I., Blay J.-Y., Wolf J., Raje N.S., Diamond E.L., Hollebecque A. (2015). Vemurafenib in Multiple Nonmelanoma Cancers with BRAF V600 Mutations. N. Engl. J. Med..

[139] Lin L., Asthana S., Chan E., Bandyopadhyay S., Martins M.M., Olivas V., Yan J.J., Pham L., Wang M.M., Bollag G. (2014). Mapping the molecular determinants of BRAF oncogene dependence in human lung cancer. Proc. Natl. Acad. Sci. USA.

[140] Okimoto R.A., Lin L., Olivas V., Chan E., Markegard E., Rymar A., Neel D., Chen X., Hemmati G., Bollag G. (2016). Preclinical efficacy of a RAF inhibitor that evades paradoxical MAPK pathway activation in protein kinase BRAF-mutant lung cancer. Proc. Natl. Acad. Sci. USA.

[141] Montagut C., Sharma S.V., Shioda T., McDermott U., Ulman M., Ulkus L.E., Dias-Santagata D., Stubbs H., Lee D.Y., Singh A. (2008). Elevated CRAF as a potential mechanism of acquired resistance to BRAF inhibition in melanoma. Cancer Res..

[142] Paraiso K.H.T., Xiang Y., Rebecca V.W., Abel E.V., Chen Y.A., Munko A.C., Wood E., Fedorenko I.V., Sondak V.K., Anderson A.R.A. (2011). PTEN loss confers BRAF inhibitor resistance to melanoma cells through the suppression of BIM expression. Cancer Res..

[143] Tsamis I., Gomatou G., Chachali S.P., Trontzas I.P., Patriarcheas V., Panagiotou E., Kotteas E. (2022). BRAF/MEK inhibition in NSCLC: Mechanisms of resistance and how to overcome it. Clin. Transl. Oncol..

[144] Baik C.S., Myall N.J., Wakelee H.A. (2017). Targeting BRAF-Mutant Non-Small Cell Lung Cancer: From Molecular Profiling to Rationally Designed Therapy. Oncologist.

[145] Nazarian R., Shi H., Wang Q., Kong X., Koya R.C., Lee H., Chen Z., Lee M.-K., Attar N., Sazegar H. (2010). Melanomas acquire resistance to B-RAF(V600E) inhibition by RTK or N-RAS upregulation. Nature.

[146] Villanueva J., Vultur A., Lee J.T., Somasundaram R., Fukunaga-Kalabis M., Cipolla A.K., Wubbenhorst B., Xu X., Gimotty P.A., Kee D. (2010). Acquired resistance to BRAF inhibitors mediated by a RAF kinase switch in melanoma can be overcome by cotargeting MEK and IGF-1R/PI3K. Cancer Cell.

[147] Prahallad A., Sun C., Huang S., Di Nicolantonio F., Salazar R., Zecchin D., Beijersbergen R.L., Bardelli A., Bernards R. (2012). Unresponsiveness of colon cancer to BRAF(V600E) inhibition through feedback activation of EGFR. Nature.

[148] Ahronian L.G., Sennott E.M., Van Allen E.M., Wagle N., Kwak E.L., Faris J.E., Godfrey J.T., Nishimura K., Lynch K.D., Mermel C.H. (2015). Clinical Acquired Resistance to RAF Inhibitor Combinations in BRAF-Mutant Colorectal Cancer through MAPK Pathway Alterations. Cancer Discov..

[149] Heidorn S.J., Milagre C., Whittaker S., Nourry A., Niculescu-Duvas I., Dhomen N., Hussain J., Reis-Filho J.S., Springer C.J., Pritchard C. (2010). Kinase-dead BRAF and oncogenic RAS cooperate to drive tumor progression through CRAF. Cell.

[150] Poulikakos P.I., Zhang C., Bollag G., Shokat K.M., Rosen N. (2010). RAF inhibitors transactivate RAF dimers and ERK signalling in cells with wild-type BRAF. Nature.

[151] Degirmenci U., Yap J., Sim Y.R.M., Qin S., Hu J. (2021). Drug resistance in targeted cancer therapies with RAF inhibitors. Cancer Drug Resist.

[152] Dantoing E., Piton N., Salaün M., Thiberville L., Guisier F. (2021). Anti-PD1/PD-L1 Immunotherapy for Non-Small Cell Lung Cancer with Actionable Oncogenic Driver Mutations. Int. J. Mol. Sci..

[153] Murtaza M., Dawson S.-J., Tsui D.W.Y., Gale D., Forshew T., Piskorz A.M., Parkinson C., Chin S.-F., Kingsbury Z., Wong A.S.C. (2013). Non-invasive analysis of acquired resistance to cancer therapy by sequencing of plasma DNA. Nature.

